# Supplementary material for: Contrasting Dynamics of Littoral and Riparian Reed Stands within a Wetland Complex of Lake Cerknica
Source: Plants (Basel). 2023 Feb 22;12(5):1006. doi: 10.3390/plants12051006 (PMC10005669; doi:10.3390/plants12051006)

Table S1 – Equations of fits

Equations of fits for both reed stands in all observed seasons. For every equation R-squared is provided along with p-values of constants *a* and *b*.

Location	Season	Model equation	R^2	*a* p-value	*b* p-value
Riparian stand Gorenje Jezero	2017	$y = 0.439 + 0.417*(1-e^{-0.071*x})$	0.996	1.89E-09	7.13E-03
	2018	$y = 0.311 + 0.555*(1-e^{-0.087 *x})$	0.996	1.45E-04	1.96E-02
	2019	$y = 0.304 + 0.554*(1-e^{-0.044*x})$	0.994	1.57E-10	2.19E-03
	2020	$y = 0.338 + 0.540*(1-e^{-0.047*x})$	0.987	5.03E-05	7.18E-03
	2021	$y = 0.392 + 0.482*(1-e^{-0.032*x})$	0.886	3.80E-08	1.09E-04
Littoral stand Zadnji kraj	2017	$y = 0.231 + 0.637*(1-e^{-0.027*x})$	0.973	5.76E-07	2.16E-03
	2018	$y = 0.209 + 0.539*(1-e^{-0.041*x})$	0.993	1.50E-04	4.06E-03
	2019	$y = 0.228 + 0.666*(1-e^{-0.039*x})$	0.974	4.21E-07	3.46E-04
	2020	$y = 0.263 + 0.386*(1-e^{-0.026*x})$	0.834	1.41E-02	1.39E-03
	2021	$y = 0.296 + 0.518*(1-e^{-0.031*x})$	0.956	2.21E-06	2.26E-03

Figure S1

A – 2017 growing season

Environmental data (temperatures and water level fluctuations) along with models for both reed stands in 2017. Matching x-axis provides a better visual explanation of the effect of environmental parameters on NDVI values.

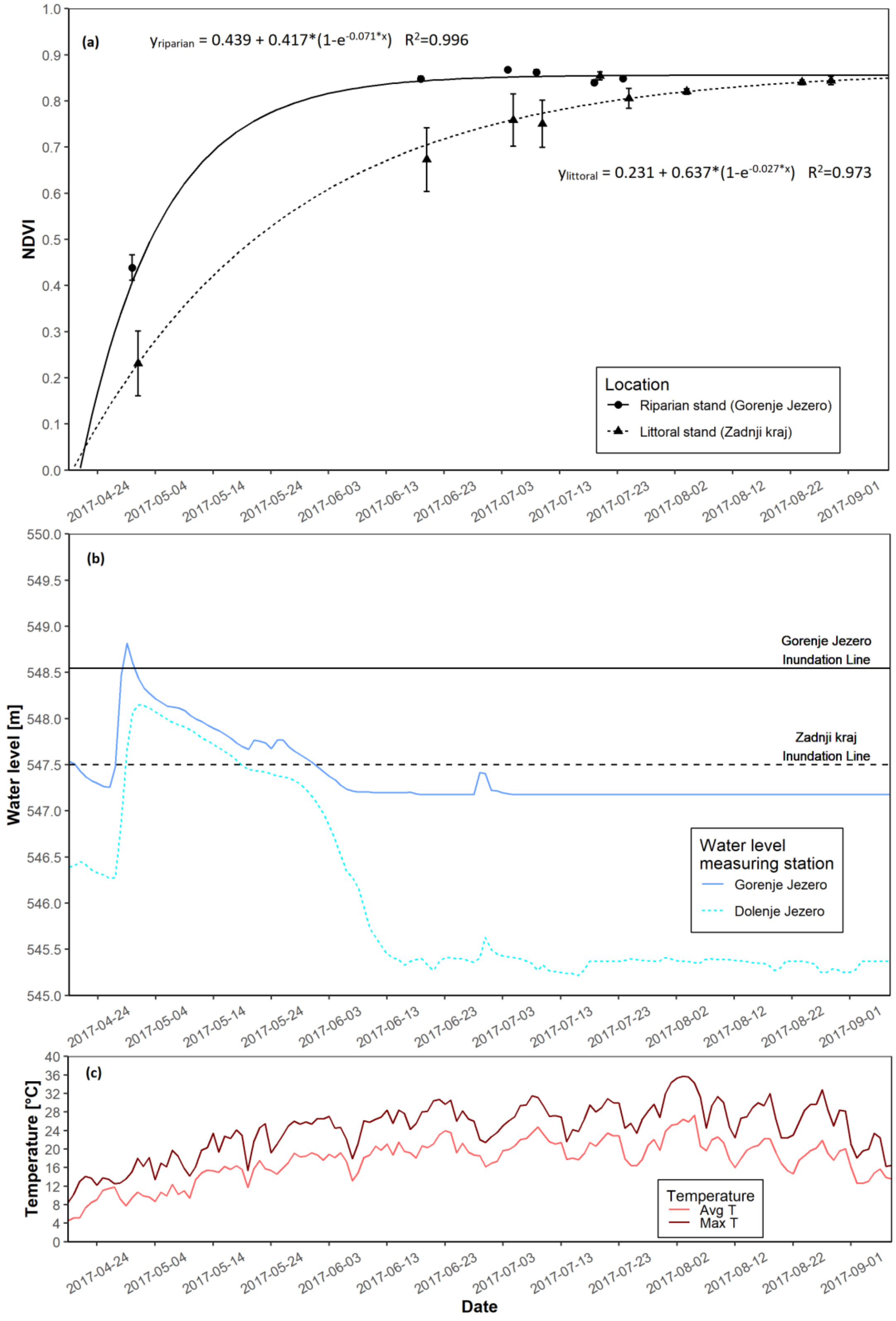

B – 2018 growing season

Environmental data (temperatures and water level fluctuations) along with models for both reed stands in 2018. Matching x-axis provides a better visual explanation of the effect of environmental parameters on NDVI values.

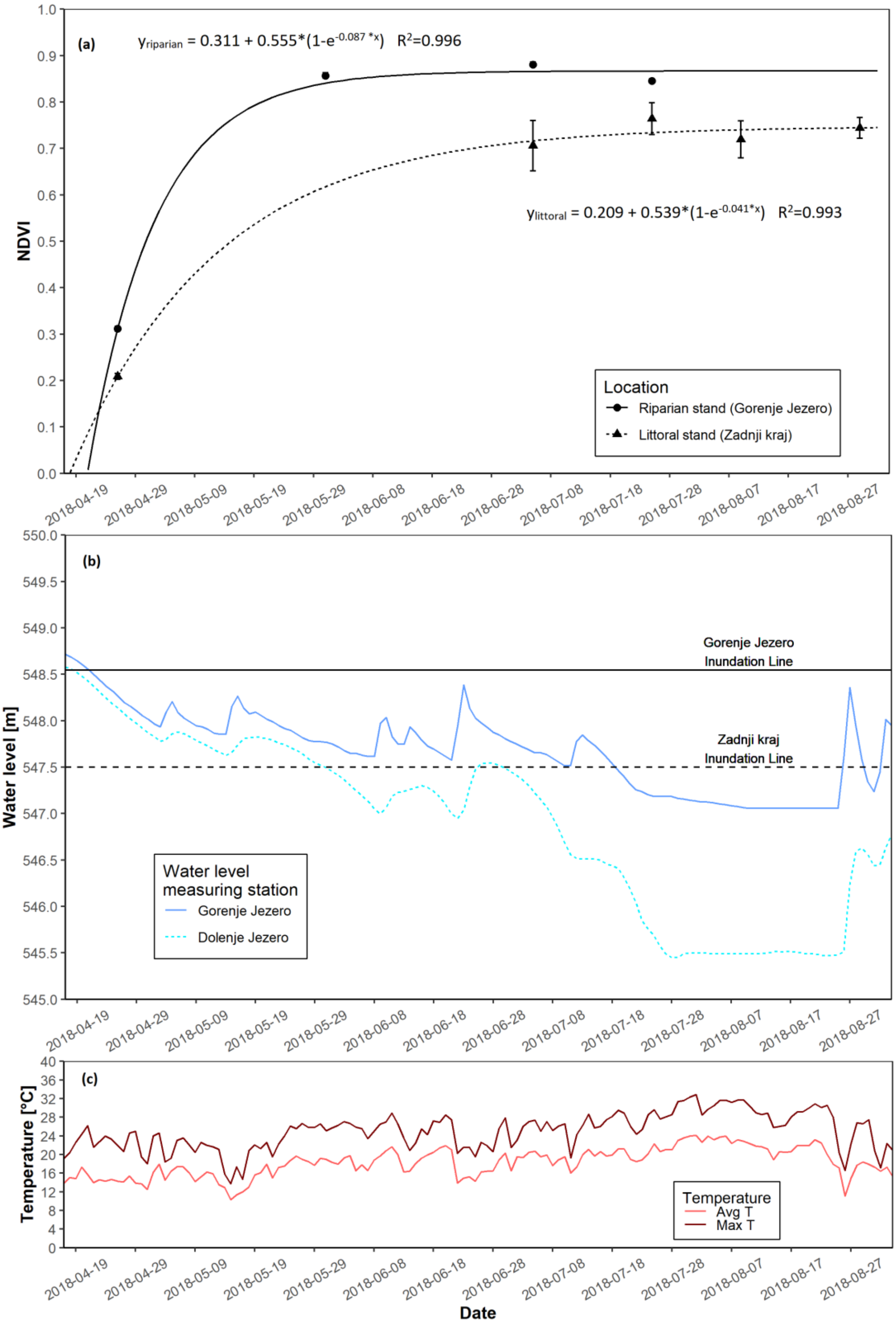

C – 2019 growing season

Environmental data (temperatures and water level fluctuations) along with models for both reed stands in 2019. Matching x-axis provides a better visual explanation of the effect of environmental parameters on NDVI values.

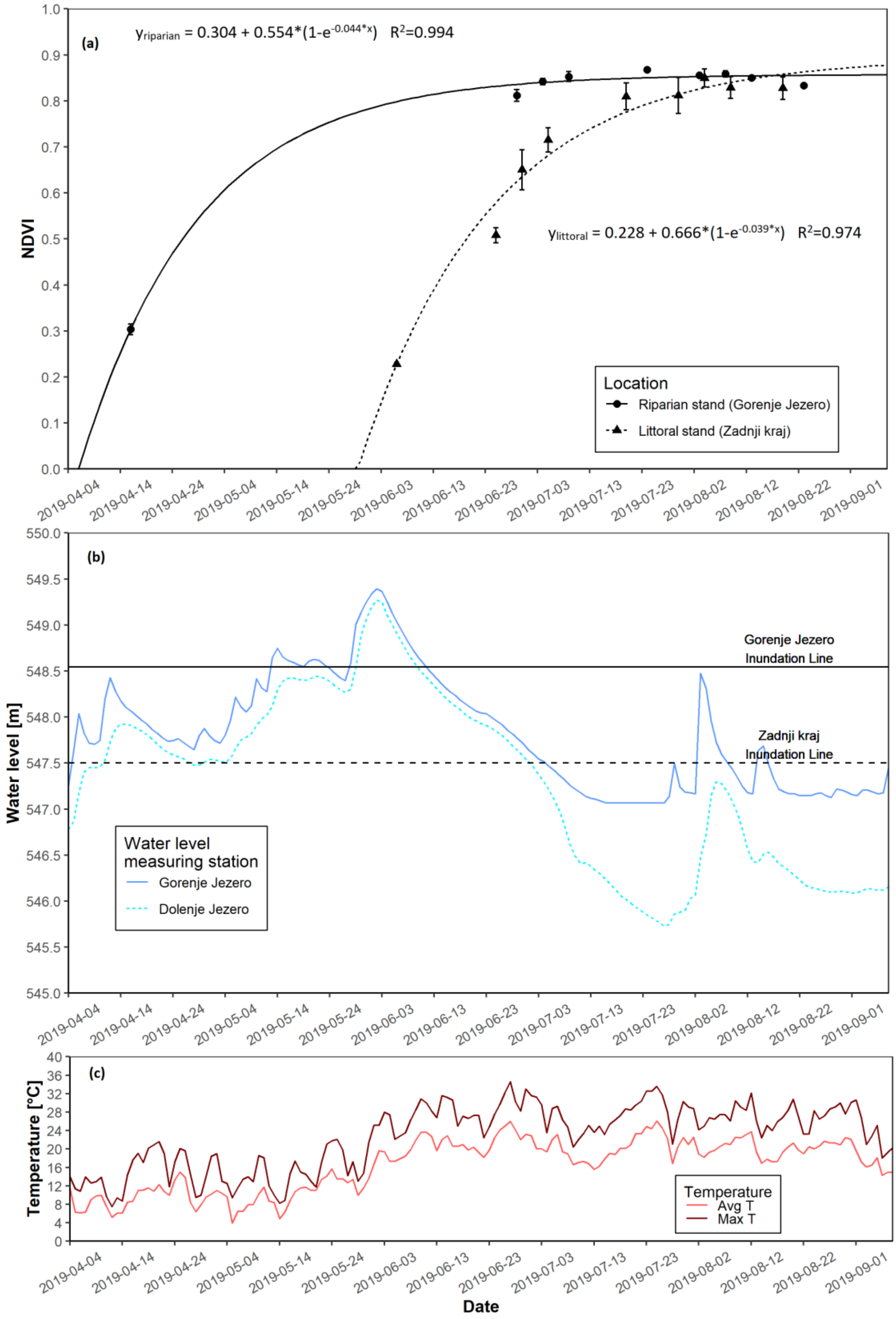

D – 2020 growing season

Environmental data (temperatures and water level fluctuations) along with models for both reed stands in 2020. Matching x-axis provides a better visual explanation of the effect of environmental parameters on NDVI values.

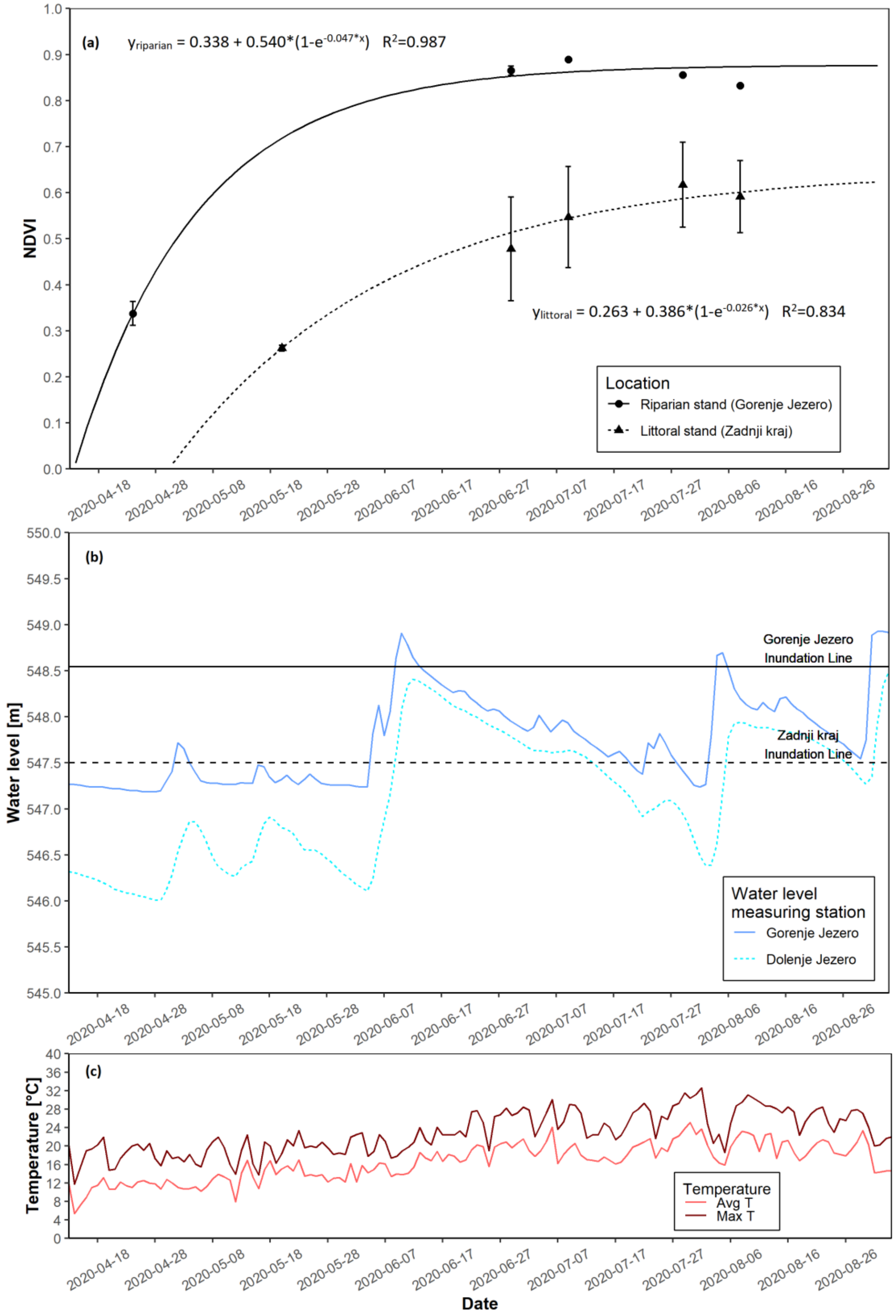

E – 2021 growing season

Environmental data (temperatures and water level fluctuations) along with models for both reed stands in 2021. Matching x-axis provides a better visual explanation of the effect of environmental parameters on NDVI values.

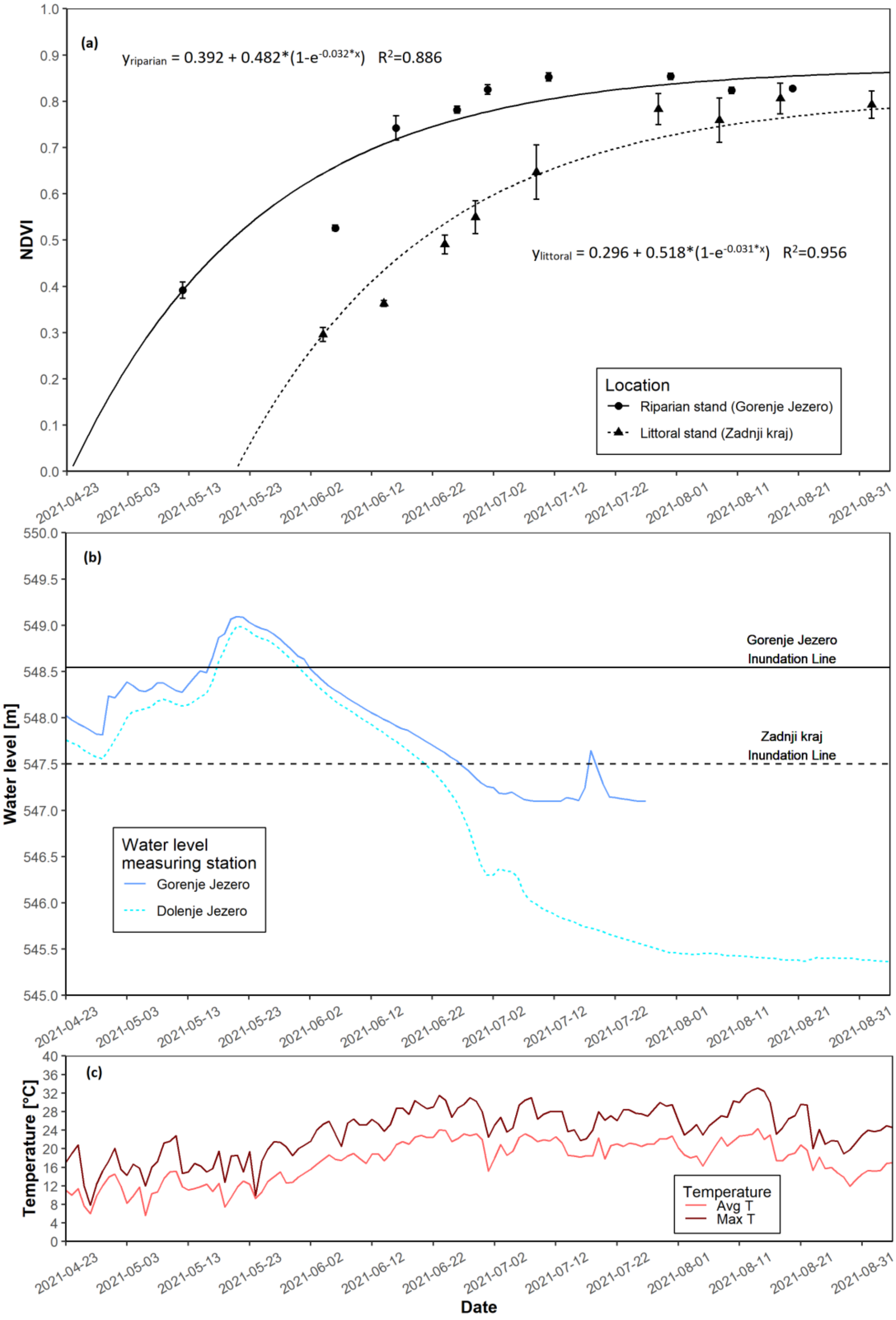

Supplement: Supplementary file 1 [file plants-12-01006-s001.zip › plants-2051012-supplementary.pdf]
